# Editorial: Therapeutic Index for Nutraceuticals in Inflammation-Related Diseases: Efficacy, Bioavailability, Metabolism and Interactions With Drugs

**DOI:** 10.3389/fphar.2020.00061

**Published:** 2020-02-13

**Authors:** Ilaria Peluso, Maura Palmery

**Affiliations:** ^1^Research Centre for Food and Nutrition, Council for Agricultural Research and Economics (CREA-AN), Rome, Italy; ^2^Department of Physiology and Pharmacology “V. Erspamer,” La Sapienza University of Rome, Rome, Italy

**Keywords:** phytochemicals, microbiota, cancer, oxidative stress, polypharmacy

It has been suggested that some plant-derived phytochemicals from food or medicinal herbs could be therapeutically effective agents in the prevention and/or management of cancer and noncommunicable diseases associated with oxidative stress and inflammation.

In this issue, the anti-inflammatory effect of plant ingredients has been investigated by Balkrishna et al., Cui et al., and Ibrahim et al. Balkrishna et al. reported anti-inflammatory and antipsoriatic effects of a sea buckthorn oil (SBKT) derived from the fruit pulp of *Hippophae rhamnoides* in the carrageenan-stimulated rat paw edema model and in the 12-O tetradecanoyl phorbol 13-acetate (TPA) stimulated psoriasis-like lesion mice model, respectively. Cui et al. observed an attenuation of the lipopolysaccharide (LPS)-induced duodenum histopathology changes in the mice treated with *Gardenia jasminoide*s decoction. On the other hand, Ibrahim et al. investigated the nephroprotective efficacy of the two pure compounds benzyl isothiocyanate (BITC) and resveratrol (RES), as well as their combination, against cisplatin-induced acute renal injury in mice. The treatment with BITC and RES combination had the higher nephroprotective effects compared to both BITC and RES alone. In these studies, in addition to the reduction of inflammatory cytokines [interleukin (IL)-1, IL-6, IL-8 and/or tumor necrosis factor-α], authors also observed inhibitions of the nuclear factor-kappa B (NF-κB) (Balkrishna et al., Cui et al.) and improvement of the total antioxidant capacity (Cui et al.), superoxide dismutase (SOD) (Cui et al., Ibrahim et al.), catalase (CAT) (Ibrahim et al.), and glutathione peroxidase (GSH-Px) (Cui et al., Ibrahim et al.).

Patti et al. reported the antioxidant activity of *Mentha longifolia L*. crude extract *in vitro* and the antiproliferative effects on two adrenocortical tumor cell models. On the other hand, Bortolotti et al. pointed out that most existing studies on *Momordica charantia* (commonly called bitter melon) bioactive compounds were performed only on cell lines and in animal models and that clinical studies are needed to establish its efficacy and safety in patients. Besides, both *in vitro* and *in vivo* studies demonstrated that bitter melon may also elicit toxic or adverse effects under different conditions and few papers discuss the anti-inflammatory and anticancer properties. Moreover, in a systematic review, Medic et al. concluded that despite anthocyanins-enriched diets proved to be effective in experimental murine models of colorectal cancer, no effect in human studies was observed. A hypothesis for the different effect in murine models and human could be the relationship among nutraceuticals and microbiota (Vamanu). From that, personalized health care for chronic noncommunicable diseases that impact quality of life should consider gut microbiota in addition to genetic and epigenetic factors, health status, polypharmacy, and moods (Sciarra et al.).

## Author Contributions

All authors listed have made substantial, direct, and intellectual contribution to the work, and approved it for publication.

## Conflict of Interest

The authors declare that the research was conducted in the absence of any commercial or financial relationships that could be construed as a potential conflict of interest.

